# Lack of transparency in clinical trials: a call for action

**Published:** 2013-12-31

**Authors:** Jorge H Ramírez

**Affiliations:** Department of Physiological Sciences, Universidad del Valle jorge.h.ramirez00@gmail.com

The purposes of pharmacological and non-pharmacological medical interventions are maintenance of good health, delay pathology and rehabilitate patients with disability. Because any intervention have the risk of adverse effects, physicians have to weigh benefits against harms before deciding the best treatment for the patient condition, a practice of rational prescription supported by high-quality evidence from randomized clinical trials and meta-analysis. Non-rational prescription of therapeutic interventions could result in severe adverse reactions. These unwanted effects could lead to increased patient visits to emergency rooms, prolonged hospitalizations, disability, loss of productivity days and patient death. Adverse reactions increase health care system costs and often lead to legal actions against physicians, hospitals, and pharmaceutical companies. 

The evaluation of scientific evidence supporting the use of medical treatments interventions depends on the existence of clear, complete and transparent descriptions of the study design, patient recruitment processes, statistical methods used for data analysis, results and conclusions. Prospective registration of clinical trials (*i.e.:* before the recruitment of the first patient) in a public registry (*e.g. *ClinicalTrials.gov and controlled-trials.com) is a necessary process to improve the transparency of interventional studies. Disclosure of agreements between the sponsor and the principal investigators, restricting in some way the publication of the study results, should also be appropriately informed in the process of the study registration. Authors must also provide a declaration of competing interests during the publication of the study. 

For this purpose, the International Committee of Medical Journal Editors (ICJME) have a standard conflict of interests form: http://www.icmje.org/coi_instructions.html. 

Availability of complete and accurate data of drug effects is necessary to prevent severe adverse drug reactions through the improvement of doctors prescription practices, the appropriate design of health-care system policies and the effective implementation of pharmacological surveillance programs.

## Registration and reporting quality of clinical research

Approximately ten years ago the ICMJE published the statement for mandatory registration of clinical trials^1^. The original intention of clinical trials registration was to improve transparency in clinical trials. Since the publication of this statement it has been demonstrated that such requirements are frequently ignored by medical journals around the world^2^. Prospectively registration (*i.e. *before the recruitment of the first patient) is recommended to improve transparency, to track changes in the study protocol, to avoid retrospective registration of selected studies, and to identify trials not published for several reasons (*e.g.* negative results, violations of the study protocol, adverse events, among others). 

The Consolidated Standards for Reporting Clinical Trials (CONSORT) were published in 2001 with the intention to improve the quality of reporting clinical trials in medical journals. A large number of interventional studies published in indexed medical journals do not comply with these guidelines^2^ ^,^ ^3^. Nowadays, these requirements are absent in a considerable number of peer-review medical journals around the world. The CONSORT were originally published for parallel group clinical trials with subsequent extensions added according to the different types of study the clinical trial design (*i.e. *cluster trials, non-inferiority and equivalence trials, and pragmatic trials), interventions (*i.e. *herbal medicinal interventions, non-pharmacological treatment interventions, and acupuncture) Data extensions have been published by CONSORT to improve descriptions of patient-reported outcomes, harms, and abstracts. Researchers are strongly advised to have an appropriate understanding of these guidelines to successfully accomplish a clinical trial in every phase (*i.e.* planning, design, enrollment, data analysis and disclosure of results). Recently, there is a CONSORT update available^4^. 

Only one out of five published clinical trials by Latin American and Caribbean journals reported a registration number. Author's information about CONSORT is currently provided only by 13% of these medical journals^2^. The Latin-American Ongoing Clinical Trials Registry (LATINREC) is not operating despite the efforts of many people involved in their creation and promotion^5^. The impact of unavailable clinical trials records is a major barrier in the process of data gathering for systematic reviews and meta-analysis. A recent cross-sectional study published in British Medical Journal found that 29% of registered phase III clinical trials, involving more than 500 patients, are still unpublished after more than five years of study completion^6^. Percentage of unpublished clinical trials in this study was higher among industry funded (32%) than other types of sponsors (18%). In addition, almost 8 out of 10 of the unpublished trials had no results available at ClinicalTrials.gov.

## Is data obtained from clinical studies in Colombia sufficiently shared?

**Figure 1:**
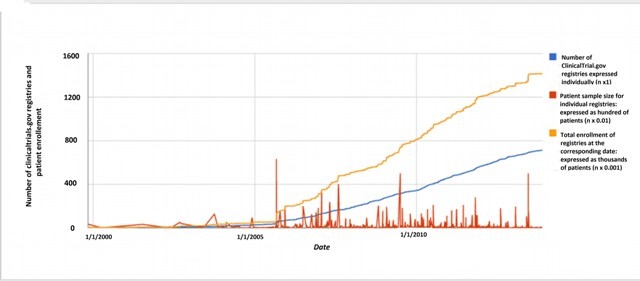
Chronological information of ClinicalTrials.gov registries and patient enrolment in Colombia.

According to ClinicalTrials.gov there are 712 studies registered for Colombia as a location for patient recruitment (Data search: December 5 2013). Most of these trials were designed to evaluate the safety and effectiveness of approved drugs in off-label conditions in postmarketing studies (phase IV) and to test investigational new drugs (clinical trials, phases one to three). The majority of ClinicalTrials.gov studies are multicentric phase III clinical trials, with patients are recruited in Colombian cities as well as other locations around the world. Approximately four out of ten studies registered in this clinical trial platform are not published after three years or more of the study completion date (Table 1). Colombian ClinicalTrials.gov records (n= 712) have data of more than 1,400,000 patients, which have been recruited in Colombian cities, as well as other locations around the world in the case of multicentric studies (Fig. 1). More than 30% of these studies were retrospectively registered (after the beginning of patient enrollment). ClinicalTrials.gov registries are frequently published more than one time, increasing the amount of visible scientific evidence for interventions and fragmenting data of one study into multiple papers, a practice known as 'salami slicing'. Approximately two papers are published for each ClinicalTrials.gov record sponsored by the pharmaceutical industry in Colombia (Table 1). In some instances, data from large studies could not be reported in one single paper and multiple publications are legitimate, in such cases authors should cite the primary study register and previously published papers. 

**Table 1:**
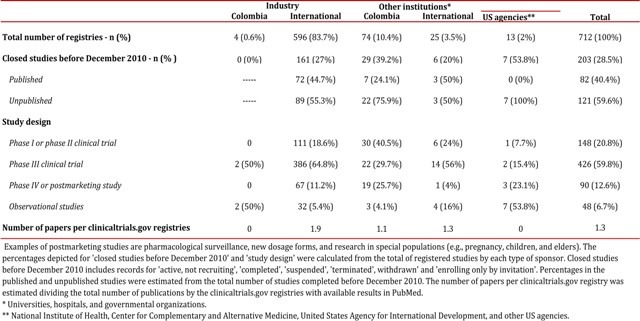
Characteristics of clinicaltrials.gov records in which Colombian institutions are listed as locations for patient recruitment.

## Consequences of human studies with unpublished results: what can be done?

Data from clinical trials with widely prescribed drugs have not been published. Pregabalin, gabapentin, paroxetine, oseltamivir, zanamivir are only some examples of drugs in which Phase III trials remain unpublished several years after study completion. Unpublished clinical trials could result in negative consequences in the economy of healthcare systems and mislead therapeutic decisions in clinical practice. Oseltamivir (Tamiflu ®) is only one example in which unpublished data resulted in unfortunate decisions by policy makers around the world. Oseltamivir was approved for the treatment of influenza by the European Medications Agency (EMEA), the Food and Drug Administration (FDA), and the National Institute for the Surveillance of Medicines and Foods (INVIMA) in Colombia. Tamiflu was also recommended by The World Health Organization (WHO) and stockpiling of this drug was encouraged by the Centers for Disease Control and Prevention (CDC). During 2009 the Ministry of Social Protection from Colombia acquired more than 1 million doses of oseltamivir at a total price of $34,000 millionm colombian pesos (approximately $16 millions USD).^7^ Most doses of oseltamivir were never used and passed their expiration date. Agencies through the world did not review the full Tamiflu dataset before the publishing statements supporting recommendations encouraging their use, purchase, and stockpiling of this drug. To the present day, results for the majority of Roche's Phase III intervention trials for oseltamivir remains unpublished over one decade after their completion date. 

A recent investigation report describing ethical violations of industry-sponsored clinical trials in developing and emerging economies (Argentina, Russia, Ukraine, and India) was recently published by the Berne Declaration^8^. Ethical violations such as abusive use of placebo, inappropriate informed consent, absence of compensation in patients affected with serious adverse events and unavailable access for participating patients to medications in the post-trial period were identified in the 4 country reports. Recently, the FDA issued a regulation in which data from clinical trials have to be disclosed after one year of the study completion date, a requirement that hopefully will improve disclosure of data obtained from clinical research. The experience obtained with the implementation of this new requirement by the FDA should be analyzed by other drug regulatory agencies around the world. 

Administration of healthcare interventions is currently supported by a small number of clinical trials, preferentially published due to their positive results, and important data is hidden to physicians, patients, healthcare policy makers, and the general public^9^. A recent article analyzed the negative impact of the Trans-Pacific partnership on drug prices and data access^10^. Transparency of clinical trials, rights for open data, and strengthening of ethical committees should be appropriately discussed between the participating nations of this agreement. 

An international campaign to restore invisible and abandoned trials is an initiative proposed by the Cochrane Collaboration, the British Medical Journal, PLOS publishers, Bad Science, the Centre for Evidence Based Medicine, The Dartmouth Institute for Public Policy & Health Practice, Sense About Science, and The James Lind Alliance. Several institutions around the world have signed the All Trials petition to restore abandoned and unpublished trials but only the Drug Information Center of the National University of Colombia (CIMUN) is included among Colombian institutions. Some Medical Journals are taking further actions to ensure clinical trials data access.

For example, starting in January 2013 the British Medical Journal (BMJ) will no longer published any clinical trial in which authors do not compromise, upon reasonable request, to make relevant anonymized patient level data accessible. Information about these initiatives could be found in the following webpages: www.alltrials.net and www.bmj.com/open-data.

Increasing the number of visits to primary care physicians and improving patient adherence to prescriptions are crucial objectives in public health and healthcare systems. Public trust in medicine is essential to achieve high standards in the provision of medical services, confidence of patients in doctors could be seriously threatened in a scenario in which the beneficial effects of interventions are selectively reported, whereas severe adverse reactions are hidden from the public eye.

 Latin American Journals should consider the support of initiatives to restore abandoned and unpublished clinical trials, request the authors commitment to make anonymized patient level data available, and strengthen the requirements for clinical trial registration and appropriate guidelines (*i.e.,* CONSORT, STROBE, and PRISM).

## Methods for identifying registries and results of human studies in Colombia

Registered studies were identified by visualization of ClinicalTrials.gov registries on map. Selected registries for the analyses presented in this paper included the participation of Colombian centers in patient recruitment. Twenty-one available fields for the eligible study records (n= 712 registries - December 5 2013) were downloaded from ClinicalTrials.gov as a plain text archive.

Identification of scientific publications for closed study records (see definitions on: http://clinicaltrials.gov/ct2/help/how-find/advanced/field-defs) completed before December 2010 (n= 202) was performed in two phases.

1. Initial screening using expert search terms on PubMed using the unique Number of Clinical Trial (NCT) assigned to registered studies (*e.g.,* "NCT00000001 OR NCT00000002 OR NCT...").

2. Results for studies in which publication were not identified by expert search terms in PubMed were sought in the 'publication field' available in ClinicalTrials.gov for individual records (publications linked to ClinicalTrials.gov records are not available for download). In addition, unique terms for each record (*e.g., *NCT, other study codes, and acronym) and other keywords (*e.g,* interventions, authors, title words) were used for identification of study results in the Google Scholar search engine. 

The complete database used for the analysis presented in this paper is available in the following link: http://goo.gl/aQxOmw. Comments are allowed in the spreadsheet settings and readers are invited to report any inconsistency in the database (*e.g., *missed data, incorrect values, inappropiate calculations), to independently analyze the data and to suggest ideas for data collection and analysis. The latest data for studies located in Colombia and other countries around the world is free for public consultation at the ClinicalTrials.gov webpage. 
